# PhaseXplorer Creates High-Dimensional Phase Diagrams with Closed-Loop Active Learning

**DOI:** 10.1021/acsnano.5c07268

**Published:** 2025-11-03

**Authors:** Stef A. H. Jansen, Lasse S. A. Dreyer, Jule van Basten, Yihan Yao, Daniel E. Otzen, Tom F. A. de Greef, Tuomas P. J. Knowles, E. W. Meijer, Nadia A. Erkamp

**Affiliations:** † Institute for Complex Molecular Systems (ICMS), 3169 Eindhoven University of Technology, Eindhoven 5612 AJ, The Netherlands; ‡ Interdisciplinary Nanoscience Center (iNANO), Aarhus University, Aarhus 8000, Denmark; § Yusuf Hamied Department of Chemistry, 2152 University of Cambridge, Cambridge CB2 1EW, U.K.; ∥ Cavendish Laboratory, Department of Physics, University of Cambridge, Cambridge CB3 0HE, U.K.; ⊥ School of Chemistry and RNA Institute, University of New South Wales, Sydney 2052, Australia

**Keywords:** phase separation, microfluidics, active learning, closed-loop, PhaseXplorer, phase diagram

## Abstract

Phase separation fundamentally governs material properties and cellular function on multiple organizational scales. Conventional approaches to studying this nonlinear phenomenon necessitate resource-intensive experiments. As such, investigations were limited to low-dimensional space. We present PhaseXplorer, a platform that combines microfluidics, microscopy, and machine learning to efficiently study phase separation systems. PhaseXplorer autonomously designs, generates, and analyzes samples in a closed-loop active learning workflow until an accurate phase diagram is obtained. Using an acquisition function that balances exploration and exploitation, all the phase boundaries are located with minimal sampling. A convolutional neural network executes real-time image recognition to identify microfluidic droplets and phase separation within them in less than 1 ms per droplet. PhaseXplorer standardizes analysis across experiments and does not require calibration nor extensive postexperiment analysis. We demonstrate PhaseXplorer’s capabilities using a poly rA model system by creating a four-dimensional phase diagram 100 times faster than traditional methods while simultaneously consuming 10,000 times less material.

## Graphical abstract

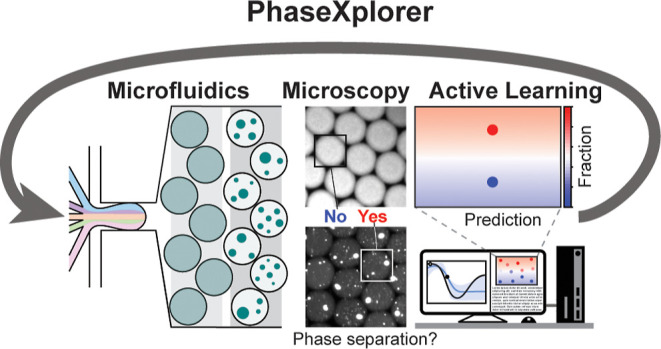

## Main

Phase separation (PS) is a fundamental mechanism underlying essential cellular functions and numerous diseases.^1,2^ Expanding mechanistic insights into PS offers prospects for disease treatment and materials science applications.^3^ However, the intrinsic complexity of these systems presents a significant challenge. Biomolecular condensates, gels, and aggregates can consist of hundreds of different molecules.^4^ Their formation depends strongly and nonlinearly on the concentration of these molecules, ionic strength, temperature, and pH.^5^ While understanding PS systems offers tremendous potential,^3,6,7^ existing experimental methods are insufficient for efficiently studying these systems in high dimensions. The current state-of-the-art combines microfluidic technology with microscopy to systematically study PS under varying conditions.^8–10^ However, the number of required data points, time, and material required to map the phase diagram via exhaustive sampling increases exponentially with each additional variable, limiting our understanding of multidimensional phase behavior.

Active learning (AL) has emerged as a computationally efficient, data-driven methodology^11–16^ to efficiently navigate high-dimensional phase diagrams by selectively targeting the most informative experiments.^17–20^ Here, we integrate microfluidic technology, microscopy, and machine learning algorithms to create a closed-loop active learning platform called PhaseXplorer. The platform operates without prior calibration or molecular-specific parameterization, requiring only solutions from which it generates samples. Automated imaging, droplet detection, and PS detection provide input for the surrogate Gaussian process regression (GPR) model, which makes a prediction that is evaluated to select the optimal samples for the next iteration. It continues to iteratively generate and analyze samples until an accurate phase diagram has been produced. We apply this platform to our model system of polyadenylic acid (poly rA). This RNA homopolymer is known to phase-separate in the presence of polyethylene glycol (PEG) and salt, creating liquid or gel-like assemblies.^21^ Notably, this system has not been studied in more than 2 dimensions per experiment. Here, we create 3D and 4D phase diagrams for this system. Creating phase diagrams with PhaseXplorer uses orders of magnitude less material and time than previously established methods and thereby enables the mapping of phase diagrams across more variables. As such, this platform will provide a versatile method for tackling complex PS challenges.

## Results and Discussion

### Closed-Loop Platform

To determine if a system will undergo phase separation (PS), the compounds of interest are mixed, and PS is detected by microscopy, scattering, or other methods.^22,23^ Typically, microliter-scale samples are prepared using micropipettes. Here, we instead use microfluidic technology to prepare picolitre samples (Figure 1a, Methods). Our active learning cycle is initiated by mixing aqueous solutions into fluorinated oil, which pinches them into small droplets. The composition of the droplets is determined by the amount of each solution added to them, which, in turn, depends on the relative flow rates of the solutions, which are controlled by the computer. Once formed, the fusion of the droplets is prevented by the surfactant added to the oil. After an incubation period, PS in the droplets can be detected. Two fluorescent dyes are added to the solution: one to locate the droplets and the other to detect the PS.

**Figure 1:**
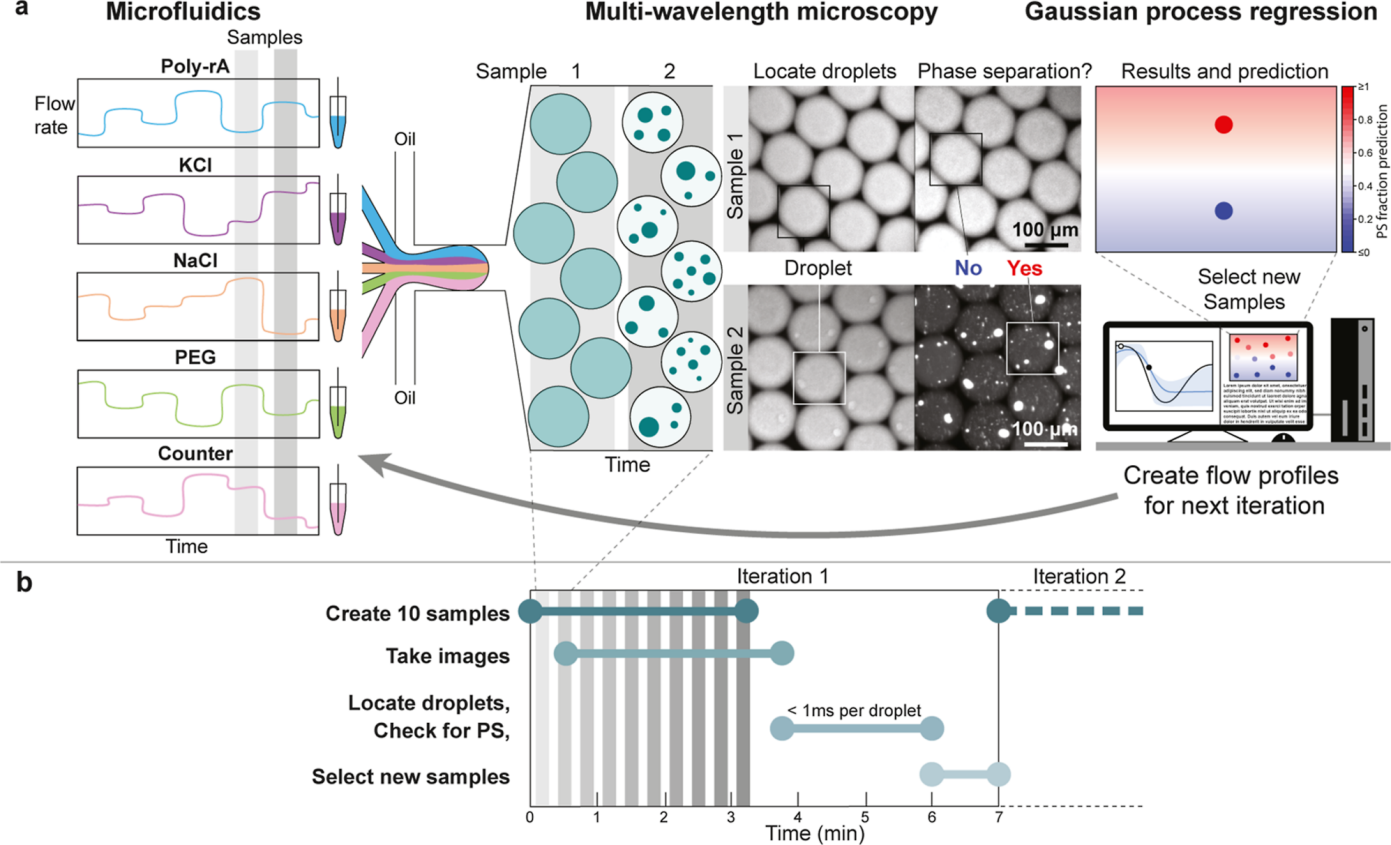
PhaseXplorer: A closed-loop active learning platform to study phase separation. (a) Microfluidics, microscopy, and machine learning are combined to study PS. For each sample, droplets are prepared, incubated, and pictured. A convolutional neural network locates droplets in pictures and checks for phase separation (PS). Based on the PS fraction of droplets for each sample, the Gaussian process regression model will predict the PS fraction of the design space. By evaluating the prediction with an acquisition function (Figure 2), the most informative samples for the next iteration are selected. These compositions are converted to flow rates, which are forwarded to the microfluidic technology. (b) Typical timeline of the experiment. Droplets are produced for 20 s per sample. The incubation time of the samples is 30 s, meaning imaging finishes 30 s after droplet creation has ended. Droplet analysis takes roughly 1 ms per droplet. Depending on the number of droplets in the pictures, the analysis of 10 samples takes around 2 min. The selection of new samples takes around a minute. In total, a typical iteration time is 7 min to design, create, and analyze 10 optimally chosen samples in multidimensional space, making it a highly efficient setup.

Droplet imaging occurs simultaneously across multiple spectral channels. For each sample, we generate about 1000 droplets (replicates) in 20 s, of which approximately 50–250 are captured within the imaging field and subsequently analyzed. The other droplets are discarded because they are produced during flow rate adjustments or because the droplets are not fully visible in the images. To analyze the images, a pretrained convolutional neural network (CNN) is used to locate droplets and identify PS, similar to those used previously^8,24^ (Figures S1 and S2 and Table S1, Supporting Methods). The PS fraction is calculated by dividing the number of phase-separated droplets by the total number of droplets detected. After all samples have been analyzed, the Gaussian process regression is the next step in the active learning loop.

Gaussian process regression (GPR) is chosen over other regression models because it yields uncertainty estimates and good interpretability. Additionally, GPR works well for this data size and has been shown to effectively locate phase boundaries in theoretical work.^17^ The GPR model constructs the probability of PS over the entire parameter space of the experiment. The model outputs a probability distribution of phase behavior, which can be used by the acquisition function (more details in the coming section) to select the next samples that provide the most information.^17,25,26^ The newly selected compositions are converted to flow rates and sent to the pressure pumps, marking the start of the next iteration. Together, these techniques create a closed-loop platform.

To assess the temporal efficiency of PhaseXplorer, we consider the duration of each step in an iteration, Figure 1b. Per iteration, we create 10 samples as this offers a reasonable balance between making use of the intelligent sample selection and keeping waiting time low. Approximately half of the iteration time is spent creating droplets for these 10 samples. A 30 s delay is introduced between droplet creation and imaging to allow enough time for PS to occur. This time can be reduced or extended depending on researcher preferences and system requirements. Once all the images have been acquired, the script sorts them into a separate folder for each corresponding sample and then starts the image analysis. Droplet identification and sorting takes the script approximately 1 ms per droplet, keeping analysis time to a minimum. New samples are selected via Gaussian process regression and subsequent minimization of the acquisition function in about a minute. None of the steps require any input from the researcher, and all are controlled by Python scripts provided. The total iteration time to design, create, and analyze 10 optimally selected samples in multidimensional space is approximately 7 min. These results demonstrate PhaseXplorer’s exceptional temporal efficiency. In comparison to other closed-loop active learning platforms,^11,12,17,27^ PhaseXplorer uses similar machine learning strategies but is the first of its kind to experimentally study phase separation in a closed-loop fashion.

### Acquisition Functions

Sample design for subsequent iterations can employ different acquisition functions.^17^ Figure 2a visualizes this process in one dimension, *x*. To initialize active learning, the first set of data points (black dots) is chosen at random. After the acquisition of these data, the Gaussian process regression predicts the PS probability. The blue line shows this average prediction, μ­(*x*), and the blue background shows the standard deviation, σ­(*x*). At the phase boundary, the PS probability is 0.5 (target). The ground truth PS fraction is shown as a dashed line. The acquisition function, α­(*x*), (bottom graph, dark blue), uses the predictive PS fraction (and target) to suggest a new data point at the minimum value of this curve (circle).

**Figure 2:**
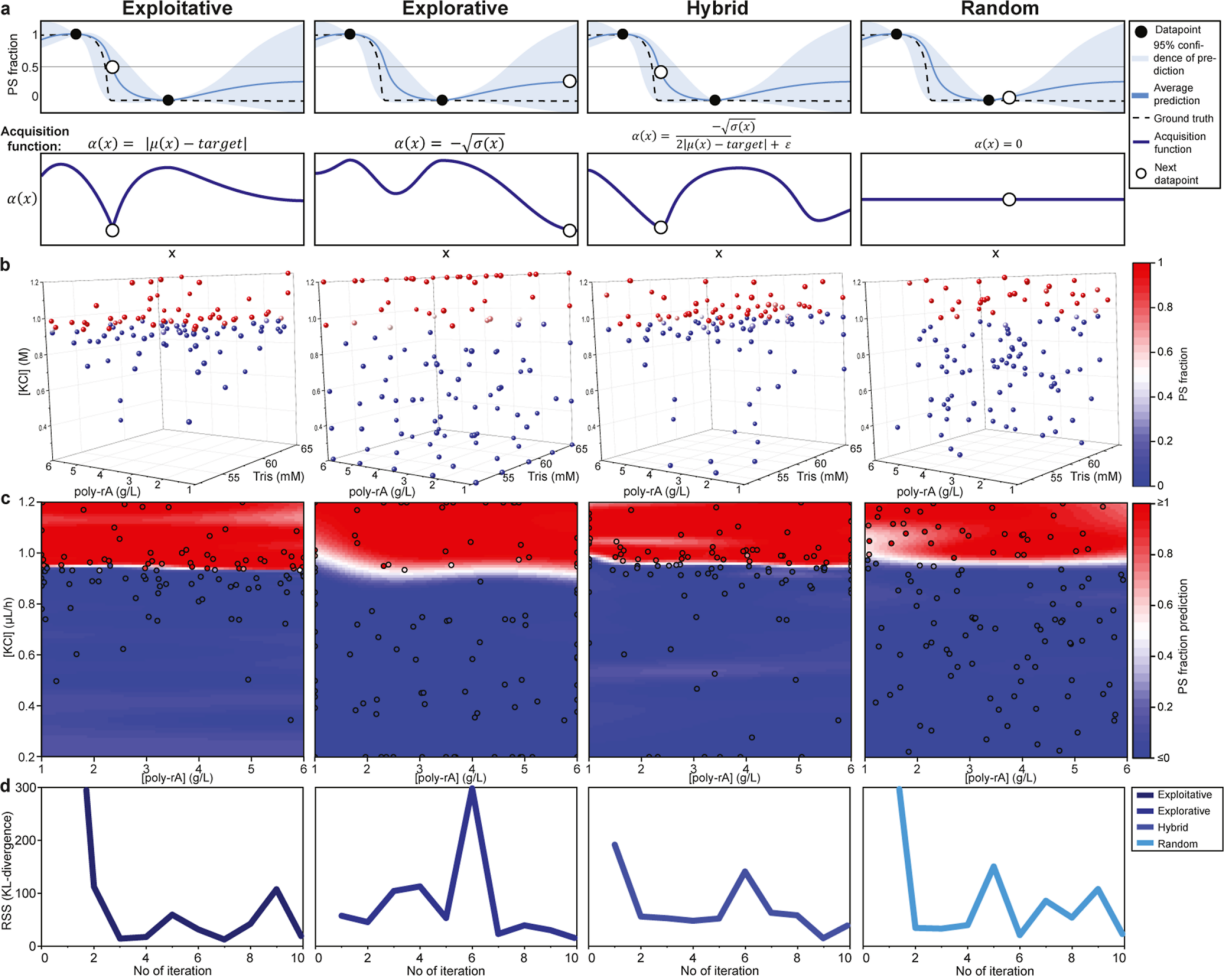
Active learning phase diagram preparation with different acquisition functions. (a) The selection of the samples of the next iteration is dependent on the acquired data and the acquisition function. Schemes with one-dimensional representations show how the acquisition functions prioritize sample selection for the exploitative, explorative, hybrid (of explorative and exploitative), and random selection of samples.^17^ From the GPR posterior predictive, μ is the mean and σ the standard deviation at data point *x*. The exploitative function selects samples where the targeted α­(*x*) value is predicted. The explorative function selects samples where the highest uncertainty (blue confidence interval) is found. Hybrid samples combine the exploitative and explorative functions. Random samples are chosen without taking the prediction or uncertainty into account. (b) 3D phase diagrams of poly rA with potassium chloride in 50 mM Tris at pH 7.5 are produced using the four acquisition functions. The sampled data points at the end of the experiments are shown, of which the color indicates the observed PS. (c) The phase diagrams are shown in 2D as a function of the poly rA and KCl solution flow rates. (d) Comparing each GPR posterior to the prior, the root sum squared Kullback–Leibler divergence of all points on a 21 × 21 × 21 discretized grid was monitored during the experiments to assess the convergence of the phase diagram prediction.

To demonstrate the effectiveness and sampling of the 4 acquisition functions, we created 3D phase diagrams of our model system using PhaseXplorer^21^ (Figure 2b). The variables were potassium chloride flow, poly rA flow, and buffer flow. The exploitative function considers the average prediction, prioritizing the samples for which the PS fraction is expected to be 0.5. We indeed observe that sampling is greatly prioritized near the phase boundary. In contrast, the explorative function selects the samples where the model uncertainty is the highest. As a result, these data points are well spaced out to reduce the uncertainty in the prediction. The hybrid function (Figures S3 and S4) combines the exploitative and explorative functions and thus locates and maps phase boundaries. As a benchmark, we also perform iterative optimization while randomly selecting data points. Random sampling is inherently inefficient because it selects many data points that are far from the phase boundary or close to the already collected data. Indeed, this approach demonstrably fails to efficiently execute either exploration or exploitation. The result of the experiment is also shown in 2D, leaving out the buffer variable (Figure 2c). The data points are shown as circles, and the predicted PS fraction is shown as the background color. A rough phase boundary is obtained for all functions. Notably, exploitative and hybrid sampling locate the “ground truth” boundary (Figure S5) more effectively. The location of the phase boundary approximates those previously observed^21^ but is here determined in greater detail.

We can monitor the convergence of the active learning experiments utilizing Kullback–Leibler (KL) divergence analysis^28,29^ (Figure 2d). Here, the KL divergence is computed between successive GP posterior predictions of the PS fraction. The KL divergence was calculated using the mean, μ, and standard deviation, σ, from the GP predictions at each point of a 21 × 21 × 21 discretized grid. We monitor the root-sum square (RSS) of these divergences over iterations. High values indicate substantial modifications to the posterior PS prediction in the last iteration. By tracking the RSS (KL divergence), we can monitor the convergence of the prediction as new data is acquired. Comparing the results for the 4 acquisition functions, we observe that the KL divergence stays relatively low for explorative, exploitative, and hybrid sampling in the final iterations, indicating convergence of the active learning experiment, but that random sampling has not finished after 10 iterations. The fast convergence for exploitative sampling may in part be because it does not screen the entire chemical space excessively, meaning that predicted values in this chemical space are unlikely to significantly change over iterations. The PhaseXplorer user is free to choose the regression function for their experiment. Taken together, these comparative analyses demonstrate that the hybrid acquisition function is the preferred option for mapping of phase boundaries, as most experimental systems require a balanced approach, both locating boundaries and comprehensively mapping them.

### Screening a Large Parameter Space

To show the ability of PhaseXplorer to navigate high-dimensional chemical space effectively, we construct a 4D phase diagram using hybrid sampling. We combined solutions to create samples with 4 variables: the flow of poly rA, KCl, NaCl, and PEG solutions (Figure 3a). PhaseXplorer performed 20 iterations of 10 samples each. During the first iterations, PS fractions near 0 or 1 are often found, while in the later iterations, more samples near the phase boundary (fraction PS = 0.5) are observed (Figure 3b).

**Figure 3:**
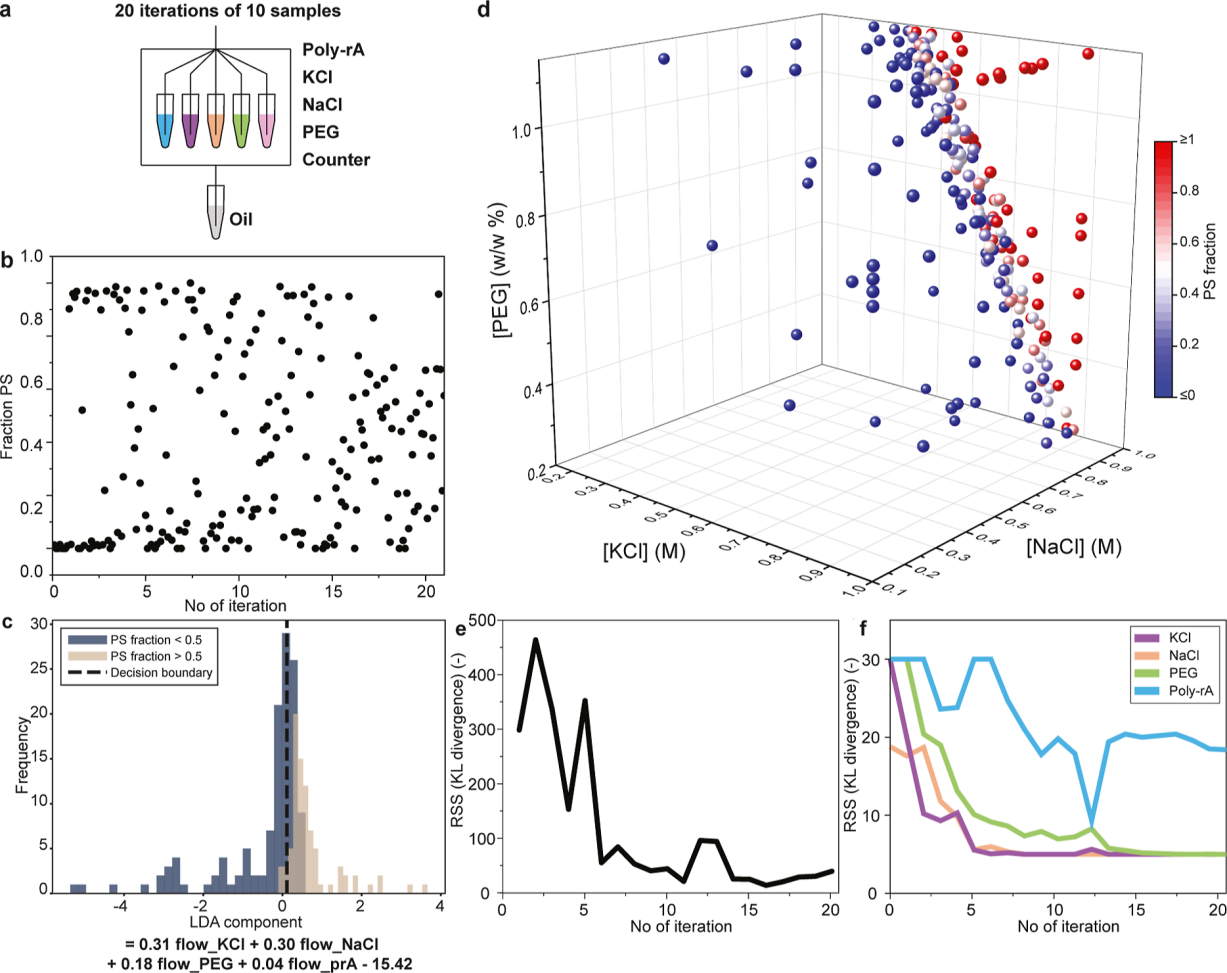
PhaseXplorer creates a 4D phase diagram. (a) PhaseXplorer performs 20 iterations of 10 samples with the hybrid acquisition function to study 4 variables: poly rA, KCl, NaCl, and PEG. (b) The observed PS fractions of all sampled data points are shown over the iterations. At the start of the experiment, the samples showed either very high or low values for the PS fraction, while the phase boundary was gradually mapped throughout the experiment, as indicated by the intermediate values of PS fractions observed. (c) To visualize the results, we perform linear discriminant analysis (LDA). We find that all 4 variables contribute positively to PS and that KCl, NaCl, and PEG are key in determining whether PS occurs. (d) 3D phase diagram projection of the 4D experiment. Data points are labeled based on their PS fraction. Indeed, the phase boundary plane (white data points) has been found. (e) The efficiency of each iteration was examined by performing a Kullback–Leibler (KL) divergence analysis on the GP predictions, monitoring the root sum squared KL divergence of each point of a 21 × 21 × 21 × 21 discretized grid. (f) The length scales in each dimension of the Matérn 5/2 kernel used in Gaussian process regression are monitored during the experiment. Lower values indicate steeper transitions in the corresponding dimension, which typically indicates that this variable influences the PS significantly. In this experiment, the poly rA concentration appeared to have the lowest effect on PS.

To visualize this 4D experiment, we reduced the dimensionality using linear discriminant analysis (LDA).^30^ This method found a linear combination of our four variables that separated the data points with and without PS. This linear combination is called the LDA component (Figure 3c). We computed the LDA component and found that there is indeed a Bayes/decision boundary (Figure S6). This is the LDA component value that separates these two data sets relatively well. Not all variables are equally important in predicting whether a sample will phase separate, as indicated by the linear coefficients of the variables in the LDA component. KCl, NaCl, PEG, and poly rA have coefficients of +0.31, +0.30, +0.18, and +0.04, respectively. The positive coefficients indicate that all variables promote PS. Given these results, we find that KCl, NaCl, and PEG appear to be the most important variables.

For qualitative assessment of the four-dimensional parameter space exploration, we plot the phase diagram against the three most influential variables (Figure 3d). Here, the data points are labeled according to the PS fraction. Indeed, data points on the phase boundary (white) are found on a curved plane that separates the low (blue) and high (red) PS fraction parts of the phase diagram. No clear boundary can be observed when placing other combinations of variables on the axis (Figure S7). From the shape of the boundary in Figure 3d and the coefficients of the LDA component, we observed that the variables contribute to PS in the order KCl > NaCl > PEG > poly rA. The trend that potassium chloride affects PS more than sodium chloride is in agreement with the ion properties^31^ and previous results in different PS systems.^32^ The positive but small effect of PEG is consistent with previous work,^5^ and the minor effect of varying poly rA concentration above the critical concentration is also expected in PS systems.^21^ These results show that the experiment has been successfully performed and that a phase boundary has been mapped in 4 dimensions.

To evaluate the quality of the experiment quantitatively, we again used the Kullback–Leibler (KL) divergence analysis^28,29^ (Figure 3e). We compare the prediction of the PS fraction of successive iterations using a 21 × 21 × 21 × 21 matrix. The root-sum-square (RSS) of these divergences varies significantly during the first iterations. After iteration 7, the prediction changed very little over time. We consider the divergence of our four variables separately (Figure 3f). Both the divergence with respect to KCl and NaCl flow stay effectively constant after iteration 5. The effect of the PEG variable is smaller, and PhaseXplorer gains information about it until approximately iteration 12. The divergence of the poly rA flow stays high and changes back and forth, particularly at iterations 12 and 13. The effect of the poly rA variable is minor, and quantifying it for longer simply adds noise to the prediction. Based on these results, we conclude that PhaseXplorer can effectively create 4-dimensional phase diagrams.

Notably, accurate phase boundary characterization was achieved prior to the full 20 iterations. Data points at the phase boundary are acquired early in the experiment (Figure 3b); the overall divergence obtains a low value after iteration 5, and all three major variables have been sufficiently investigated after iteration 12. Monitoring KL divergence during optimization allows the user to terminate the experiment automatically once the target has been obtained. In total, PhaseXplorer can perform high-dimensional experiments based on the researchers’ interest.

### Orders of Magnitude Innovation with PhaseXplorer

To highlight the innovation of PhaseXplorer, we quantitatively compare it to other methods of obtaining phase diagrams. For our calculations, we have made estimations in favor of conventional methods (Methods). Thereby, we obtain the precious material (Figure 4a) and time (Figure 4b) consumption of traditional experiments (Figure 4c), experiments using one innovation, and PhaseXplorer, which combines three innovations (Figure 4d and Tables S2 and S3).

**Figure 4:**
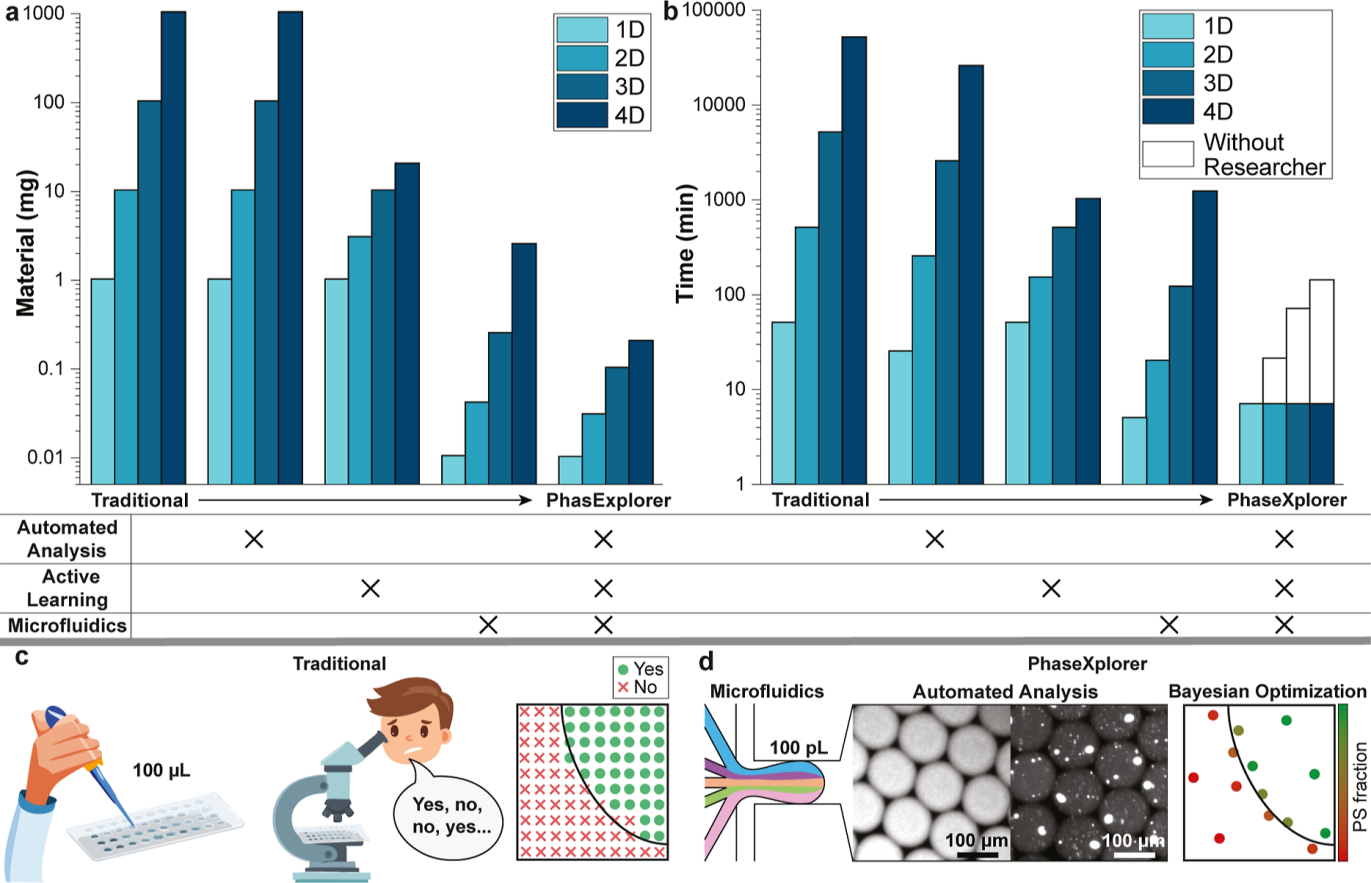
Quantitative comparison of different platforms to study phase separation. (a) Time and (b) material consumption of 1, 2, 3, and 4D PS studies performed with and without the use of microfluidics, multiwavelength imaging, and active learning. With the use of PhaseXplorer, the researcher is only present during the first iteration of the experiment, not the rest of the runtime (“Without Researcher”). Across techniques, we compare experiments that determine the phase boundary with similar accuracy, not experiments using the same number of samples (Supporting Methods and Table S1). (c) The traditional approach includes a researcher preparing samples with a micropipette. Samples are typically 100 μL, and a researcher images and analyzes samples manually. Samples can be selected in a grid-based manner. (d) PhaseXplorer combines three innovations. The microfluidics reduces the sample size by approximately 6 orders of magnitude to around 100 pL. Automatic analysis and active learning allow for continuous and efficient exploration of phase space.

In conventional experiments, samples are manually pipetted, imaged, and analyzed, taking roughly 50 min and 1 mg of precious material per 1D experiment. When automated sample analysis is applied, the material consumption remains the same, but the researcher saves experimental time. The application of active learning instead significantly reduces the amount of required material by sampling more efficiently. This allows 4D experiments to be easily designed; however, due to the large sample sizes, these larger experiments are still unrealistic for many systems. Applying microfluidics reduces sample sizes significantly.^8^ Notably, for each dimension of interest, the barcoding of droplets with a fluorescent dye may be required to distinguish different samples. Thus, the simultaneous study of 4 dimensions is usually infeasible due to spectral crosstalk. For the calculation, we assumed that one 4D experiment requires the performance of 10 3D experiments.

PhaseXplorer integrates all three key innovations to leverage the complementary technological innovations of all three. Active learning reduces the number of samples, microfluidics reduces the size of samples, and automated analysis closes the iteration loop. Thus, the researcher does not need to be present during much of the experiment (Figure 4b). Overall, PhaseXplorer performs 4D experiments with less researcher time and material than a traditional 1D experiment. Beyond this quantitative comparison, PhaseXplorer standardizes PS analysis within and across experiments since the same image recognition neural network is applied. PhaseXplorer additionally requires no calibration and automatically generates images, phase diagrams, and other information on interest. The CNN analysis is robust and works on a range of phase-separating systems (Figure S8). Notably, PhaseXplorer also has some limitations. Phase separation and droplets are detected via fluorescent signal, meaning a covalently bonded dye and barcode dye are required. Input solutions should be aqueous and have a sufficiently low viscosity to flow effectively through the chip. Nonetheless, PhaseXplorer is both a material- and time-efficient method for making phase diagrams, which excels particularly for high-dimensional experiments.

## Conclusion

Phase separation studies underlie advances in materials science, disease treatment, and our understanding of cellular behavior. Traditionally, generating phase diagrams has been time- and material-intensive. Here, we present PhaseXplorer, which integrates three technological innovations into one cohesive experimental platform. Automated analysis, active learning, and microfluidics create a closed-loop and highly efficient setup. PhaseXplorer does not require calibration, offers a choice in acquisition function, standardizes analysis, and reduces analysis time postexperiment. Compared to traditional experiments, PhaseXplorer generates 4D phase diagrams over 100 times faster and with over 10,000 times less material. We anticipate this platform will be instrumental in tackling key multidimensional challenges involving biomolecular condensates, protein-drugs, aggregation, and self-assembly.

## Methods

### Materials

Poly rA (Sigma-Aldrich, 10108626001), KCl, NaCl, PEG (average molecular mass 20.000), and HFE-7500 engineered fluid were obtained from Sigma-Aldrich. U80-FITC RNA (Biomers), RAN fluorosurfactant (RAN Biotechnologies), and Alexa Fluor 647 carboxylic acid (Thermo Fisher) were also purchased.

### Microfluidic Chip

The microfluidic chip^8^ was prepared using standard lithography techniques.^8^ Briefly, a silicon wafer mask was prepared by spinning SU-8-3025 at 48 μm height, applying 30 min of 95 °C heat, applying UV light through our mask (AutoCAD), applying 5 min of 95 °C heat, and removing excess SU-8-3025 with PGMEA cleaning. PDMS (Corning) was poured onto the mask, air was removed through a vacuum desiccator, and the PDMS was baked at 65 °C for 1 h. Biopsy punchers were used to punch holes at the inlets and outlet, and the PDMS was bonded to a glass slide by activation with oxygen plasma (30 s, 40% power, Femto, Diener Electronics). Channels were treated with 1% trichloro­(1*H*,1*H*,2*H*,2*H*-perfluorooctyl)­silane (Sigma-Aldrich) in HFE-7500, after which liquid was removed with nitrogen gas.

### PhaseXplorer Hardware

A custom confocal.nl (Figure 2) and CAIRN (Figure 3) fluorescent microscope was used with light split control for imaging at 488, 546, and 647 nm and brightfield. For this study, the 488 and 647 pictures were used. The pixel sizes are set to 0.7 (Figure 2) and 1.3 μm (Figure 3). The exposure times were 83 (Figure 2) and 10 (Figure 3) ms. Samples were prepared using Fluigent microfluidic technology equipment: pressure control (Flow EZ, 2 bar), flow units (FLU-S-D), and inner diameters 254 and 127 μm PEEK tubing.

### PhaseXplorer Software for Sample Preparation and Analysis

The Fluigent pressure pumps and flow sensors were controlled by Python using the Fluigent software development kit (SDK). During the experiment, the microscope continuously recorded images at a specified time interval. The images were sorted and automatically organized into separate folders for each sample by using Python. The convolutional neural network (CNN) was trained, and droplet analysis was performed as described fully in the Supplementary Methods section in the Supporting Information. Briefly, the images were analyzed using two CNNs: one network to recognize homogeneous droplets in an image (∼20–70 droplets) and one network to recognize PS droplets. Both networks were trained using the Yolo-NAS framework [https://zenodo.org/records/7789328].^33^ The networks were trained from scratch using the Yolo-NAS-S (small) model with an input size of (640,640). The droplet and PS CNN were trained separately for 100 epochs. The training code is available upon request. The detected droplets were filtered from erroneous droplets by shape (requiring symmetry) and radius. The image from the droplet detection channel was run through the droplet detection CNN, and the PS detection channel was run through the PS detection CNN. The detected droplets were checked for PS by comparison with the output from the PS detection CNN by a distance-to-center check, and a binary classification was recorded. To determine the PS fraction of a sample, the number of droplets with PS was divided by the total number of detected droplets.

### Active Learning Methods

To efficiently explore the formulation space, we implemented a closed-loop active learning (AL) strategy centered on a Gaussian process (GP) surrogate model. This approach enables the targeted sampling of experimental conditions that maximize information gain or objective improvement, thereby minimizing the number of required experiments and enabling the study of multidimensional formulation spaces.

#### Parameter Space and Initialization

In this study, up to four parameters of the experimental conditions (e.g., poly rA concentration, KCl concentration, NaCl concentration, and PEG concentration) were combined to define the parameter space, with user-defined limits for each variable. To initiate the AL, the first batch of experiments was randomly selected, and all subsequent batches followed the acquisition-minimization loop described below.

#### Surrogate Model

After each batch of sample preparation and data analysis, the Gaussian process regression (GPR) was used as the surrogate model, which was performed with the scikit-learn library in Python. A Matérn 5/2 kernel was combined with a constant kernel to model the smooth nonlinear response surface of the phase behavior. We selected the Matérn 5/2 kernel as it offers greater flexibility for modeling moderately rough functions compared with alternatives such as the squared-exponential kernel. To avoid overfitting, the kernel length scales were constrained within the range of each input dimension. Although the combination with a white noise kernel was initially considered, it was excluded from the final implementation, as it did not appear to improve predictive performance. The model was refitted at each iteration by using all previously collected data. To ensure robust convergence, the GP hyperparameters were optimized with up to 25 restarts of the optimizer. Generally, the GPR performs well across smooth and moderately nonlinear landscapes. The prediction of extremely sharp or discontinuous phase boundaries may require more specialized models or finer sampling.

#### Acquisition Function

To select new experimental conditions, the mean μ and standard deviation σ of the GPR posterior predictive were evaluated by the acquisition function α to select the next data points. Four acquisition functions were implemented:

- Random sampling
- Pure exploitation, which selects the samples closest to the predicted phase boundary. In this case, the target is the phase boundary with a PS fraction of 0.5.
- Pure exploration, which selects the samples with the highest model uncertainty.
- Hybrid strategy, which balances the uncertainty and proximity to the phase boundary via

$$\alpha \left(x\right)=\frac{-\sqrt{\sigma \left(x\right)}}{2\left|\mu \left(x\right)-target\right|+\varepsilon }$$

For the hybrid acquisition function, the ε variable to balance the exploitation and exploration was set at 0.5.

The acquisition functions (Figure 2a) were minimized within the user-specified variable limits using the minimize function of the SciPy library with the Nelder–Mead algorithm. The minimization of the function was initiated from 100 random initial data points, and the best minimizer was retained.

#### Batch Acquisition

To select multiple samples per iteration (in this study, typically 5–10 samples), the first sample of a batch was selected according to the minimization described above. To promote spatially diverse sampling, a Gaussian penalty function was added to the acquisition function with the maximum on the position of the previously selected batch point. The Gaussian width was adapted per dimension based on the input space range. This penalized acquisition function was then minimized to select the next batch point, after which another Gaussian function was added with its maximum at the position of the newly sampled batch point. This was repeated until a user-defined batch size was obtained.

### Calculation of Kullback–Leibler Divergence

The Kullback–Leibler divergence *D*_KL_ for two normal distributions *P* and *Q* with given means μ_1_ and μ_2_ and standard deviations σ_1_ and σ_2_ is given by

$$D_{KL}\left(P∥Q\right)=\log\left(\frac{\sigma_{2}}{\sigma_{1}}\right)+\frac{\sigma_{1}^{2}+{\left(\mu_{1}-\mu_{2}\right)}^{2}}{2\sigma_{2}^{2}}-\frac{1}{2}$$

Using the means and standard deviations of consecutive GP posteriors, the root sum of squares of the KL divergence was computed for the discretized design space without normalization of the KL divergences to preserve sensitivity to steep local transitions. In practice, active learning can be terminated once the RSS (KL) falls below a user-defined threshold and remains below this threshold for a number of iterations, indicating model convergence.

### PhaseXplorer Experimental Setup

The oil phase solution is 2% RAN fluorosurfactant in HFE-7500. All aqueous solutions contained 50 mM Tris at pH = 7.5. For the experiments in Figure 2, the solutions also contained (A) 20 g/L poly rA with 10 μM AF488, (B) 4 M KCl with 10 μM AF647, (C) an additional 50 mM Tris at pH = 7.5, and (D) no additional components (counter). For the experiments in Figure 3, the solutions also contained (A) 10 g/L poly rA with 10 μM AF488, (B) 4 M KCl with 10 μM AF647, (C) 4 M NaCl, (D) 5%w/w PEG (20k), and (E) no additional components (counter). Given these concentrations and a total aqueous flow rate of 100 μL/h, the flow rates given in Figures 2 and 3 can be easily converted to final concentrations in the samples.

### Calculations for Figure 4

For the calculations to prepare Figures 4a and 4b, we made several assumptions that were purposely done to the advantage of the non-PhaseXplorer methods. Samples for the traditional method were assumed to be 100 μL large and take 5 min to prepare, image, and analyze per sample. For a 1D experiment, we conservatively assumed the traditional method requires 10 samples including any replicates required. For each added dimension, 10 samples are added in this direction, meaning the number of samples increases by an order of magnitude for each added dimension.^34^ The “only microfluidics” condition is calculated to take similar times and volumes as reported in ref 8. Notably, 4D experiments with this setup are not possible, as fluorescent dye barcoding is required for these experiments, and spectral crosstalk becomes significant when using more than 4 dyes. As such, we have assumed that 10 3D experiments are combined to make a 4D experiment. PhaseXplorer can track how much the PS prediction changes per iteration, which is an indication of if the experiment has been completed. To be conservative about the efficiency of our platform, we have in the calculation assumed the researcher does not use this function and runs the full 11 and 21 iterations for 3D and 4D experiments, respectively. The researcher only has to be present during the first iteration to check for any errors, not for the rest of the runtime (white bars, “Without researcher”). The samples are assumed to contain the same amount of precious material per volume, 1 g/L final concentration (consistent with our experiments in Figures 2 and 3), independently of the technique. All values are reported in Tables S2 and S3.

### Further Analysis

Pictures were prepared with our Python script (provided), Fiji (2.3.051), Origin (2017), and Adobe Illustrator (29.3.1).

## Supplementary Material



## Notes

Methods on CNN training and droplet analysis; table on the held-out test on droplet and PS detection with CNN; table on precious material consumption values from Figure 4a; table on time consumption values from Figure 4a; images from the held-out test set with predicted and ground truth annotations; validation parameters of the training of droplet and PS detection CNN; images with analysis of the hybrid 3D experiment; reference diagram for the 3D experiment; further LDA analysis of the 4D experiment; projections of the 4D phase diagrams; and images of phase-separated droplet CNN analysis of samples containing various materials (PDF)
