# Supplementary material for: PhaseXplorer Creates High-Dimensional Phase Diagrams with Closed-Loop Active Learning
Source: ACS Nano. 2025 Nov 3;19(45):38981–9. doi: 10.1021/acsnano.5c07268 (PMC12632172; doi:10.1021/acsnano.5c07268)
Supplement: Supplementary file 1 [file nn5c07268_si_001.pdf]

# Supplementary information to: PhaseXplorer creates high-dimensional phase diagrams with closed-loop active learning

Stef A.H. Jansen[1], Lasse S.A. Dreyer[2], Jule van Basten[1], Yihan Yao[3], Daniel E. Otzen[2], Tom F.A. de Greef[1], Tuomas P. J. Knowles[3,4], E. W. Meijer[1,5], and Nadia A. Erkamp[1,3,*]

[1]Institute for Complex Molecular Systems (ICMS), Eindhoven University of Technology, Eindhoven, 5612 AJ, The Netherlands
[2]Interdisciplinary Nanoscience Center (iNANO), Aarhus University, Aarhus, 8000, Denmark
[3]Yusuf Hamied Department of Chemistry, University of Cambridge, Cambridge, CB2 1EW, United Kingdom
[4]Cavendish Laboratory, Department of Physics, University of Cambridge, Cambridge, CB3 0HE, United Kingdom
[5]School of Chemistry and RNA Institute, University of New South Wales, Sydney, 2052, Australia
[*]Correspondence: n.a.erkamp@tue.nl (N.A.E.)

# Contents

Supplementary Methods
Supplementary Tables 1-3
Supplementary Figures 1-8
Supplementary References

# Supplementary methods

## CNN training and droplet analysis

The two convoluted neural networks (CNN) used in this work for droplet detection and phase separation (PS) detection in the microscopy images was trained from scratch using the Yolo-NAS-S model (640x640 input size) of the SuperGradient Yolo-NAS framework ver. 3.7.1 [https://zenodo.org/records/7789328] [1] in a Python ver. 3.9.19 environment. The training of both networks were performed using standard hyperparameters and data augmentation - except for 'MixUp' augmentation. The 'MixUp' augmentation slices training data as puzzle pieces and creates unrealistic combinations. It caused the CNNs to perform poorly and was excluded. The dataset for the droplet detection CNN consisted of 3103, 560, and 187 annotated images for, respectively, training, validation, and testing. The detection of PS droplets proved to be a harder task to learn, and the dataset for the PS CNN was increased to 9243, 1730, and 577 annotated images for, respectively, training, validation, and testing. The data [2] was split randomly and is available upon request to the corresponding author. All data consisted of microfluidic droplets (PS and homogeneous) imaged on the same experimental setup used for the experiments. The data was collected and chosen to display a visually diverse set of properties in, especially, the PS droplets. The images were log-normalized, resized to the network input dimensions, and converted to 8-bit prior to training (supplementary figure 1) The test datasets were held-out and used to test performance post-training for both networks. Both networks were trained for 100 epochs and the best checkpoint was selected based on the mAP@0.5 metric.

The networks were trained 100 epochs for the droplet detection and PS detection CNN (supplementary figure 2). The mAP@0.5 and recall@0.5 all converge quickly towards unity. The recall metric shows that if there is a droplet in the image the network will find it. Precision@0.5 is farther from unity, and indicates that over the full confidence range (0.01-0.99) the networks predicts false positives. We attribute this behaviour to prediction of incomplete edge-droplets by the network. These were not annotated in the dataset, as they are undesirable for further use. Naturally, this leads to false positives. This is evident in supplementary figure 1, where both networks label droplets along the edges of the image - even though they were not part of the training data. Removal of edge-droplets was incorporated in a later part of the analysis pipeline. The evaluation of the best performing CNNs using the held-out test dataset can be seen in supplementary table 1. The confidence cutoff was set at 0.8, as this is what was used during PhaseXplorer experiments. It can be seen in the table that the precision of the CNNs recovers by raising the confidence cutoff, and the CNNs succesfully predicts the complete droplets as exemplified in supplementary figure 1 with a confidence between 0.85-0.95.

The best performing checkpoints of both CNNs were exported from the Super Gradients Yolo-NAS development framework to the 'production' framework ONNX to greatly increase the speed of execution [https://github.com/onnx/onnx]. The speed increase afforded by using a modern CNN framework compatible with a high-speed production framework such as ONNX was of importance to the development of the rest of the PhaseXplorer closed-loop active learning setup. The droplets were imaged at two fluorescent channels simultaneously during the experiments. 488 nm was used for droplet detection and 647 nm for PS detection. The respective CNNs were run on the images, and predictions with a confidence >0.6 where used for the analysis. The found droplets were further analyzed by the Python analysis script using logic checks and elementary arithmetic. The droplets were filtered for asymmetry by requiring an aspect ratio >0.8, and a diameter between 20-100 $\mu$m. Incompletely imaged droplets along the edge of the image were found and discarded by a distance check to the image edge. Finally, a distance check between droplets found using the droplet detection CNN and the PS detection CNN was used to label the droplets as either PS or not (true/false). The results (.csv files) was then handed off to the PhaseXplorer pipeline. Example images with CNN annotations from the hybrid 3D experiment are shown in supplementary figure 3 and 4.

# Supplementary Table

| Test results at confidence >0.6 | Droplet CNN | PS CNN |
|---|---|---|
| mAP@0.5 | 0.978 | 0.982 |
| Precision@0.5 | 0.825 | 0.915 |
| Recall@0.5 | 0.985 | 0.974 |
| F1@0.5 | 0.898 | 0.944 |

**Supplementary table 1. Results of the held-out test on both droplet detection and PS CNN (supplementary methods).**

| Dimensionality | Traditional | Auto-analysis | Active learning | Microfluidics | PhaseXplorer |
|---|---|---|---|---|---|
| 1D | 1 | 1 | 1 | 1 | 0.01021 |
| 2D | 10 | 10 | 10 | 3 | 0.03063 |
| 3D | 100 | 100 | 100 | 10 | 0.01208 |
| 4D | 1000 | 1000 | 1000 | 20 | 0.20417 |

**Supplementary table 2. Precious material consumption (mg) as shown in Fig. 4a.** Assumptions in the calculations and references to are given in the method section.

| Dimensionality | Traditional | Auto-analysis | Active learning | Microfluidics | PhaseXplorer |
|---|---|---|---|---|---|
| 1D | 50 | 25 | 50 | 5 | 7 |
| 2D | 500 | 250 | 150 | 20 | 21 |
| 3D | 5000 | 2500 | 500 | 120 | 70 |
| 4D | 50000 | 25000 | 1000 | 1200 | 140 |

**Supplementary table 3. Time consumption (min) as shown in Fig. 4b.** Assumptions in the calculations and references to are given in the method section. Note that the time reported for PhaseXplorer is the time required by the machine. The researcher only has to be present during the first 7 minutes of the experiment, irrespective of the duration of the experiment.

# Supplementary Figures

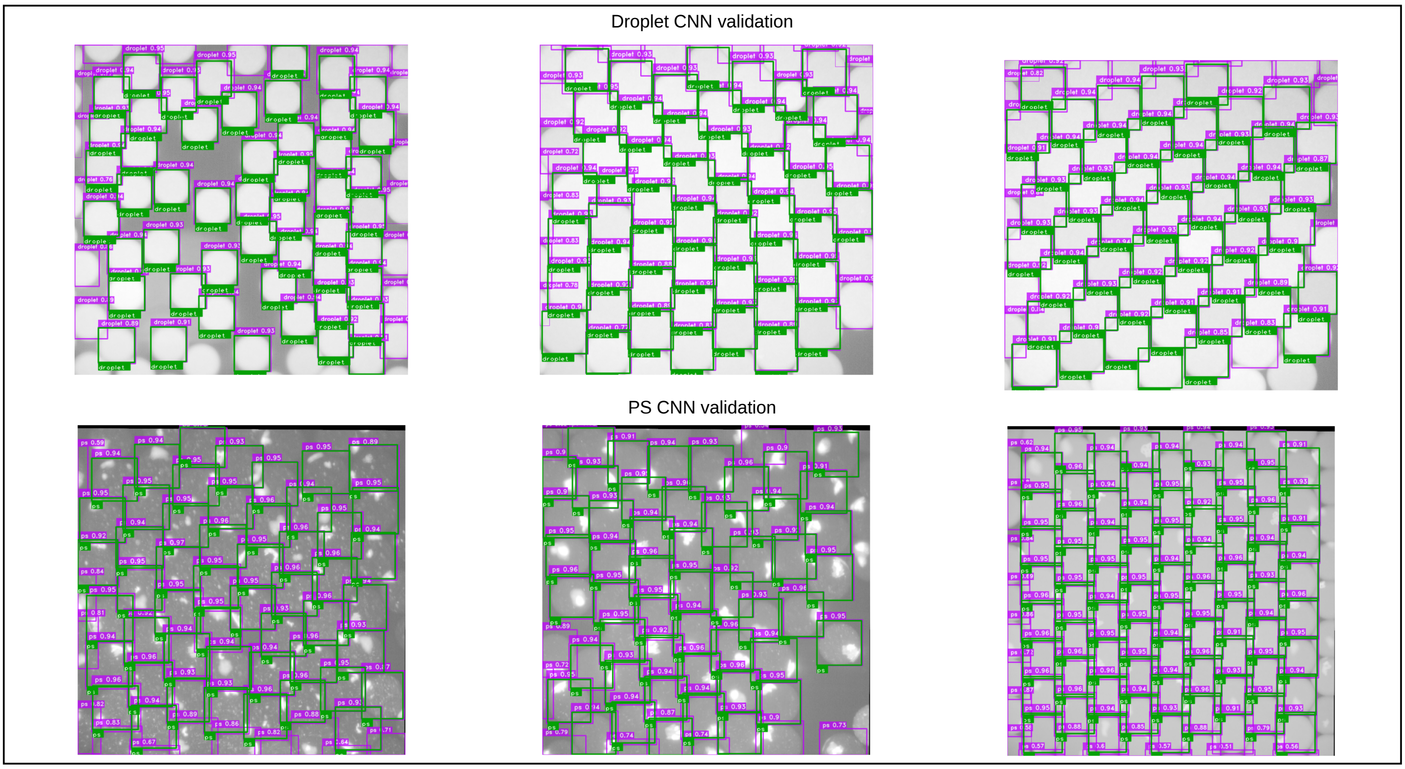


**Supplementary Figure 1. Sample images from the held-out test set with predicted and ground truth annotations**. The predictions are shown in purple, and the ground truth in green. The confidence of the predictions are shown in the label. It is exemplified that both CNNs perform well on complete center droplets. Please note that these images have been log-normalized and converted to 8-bit.

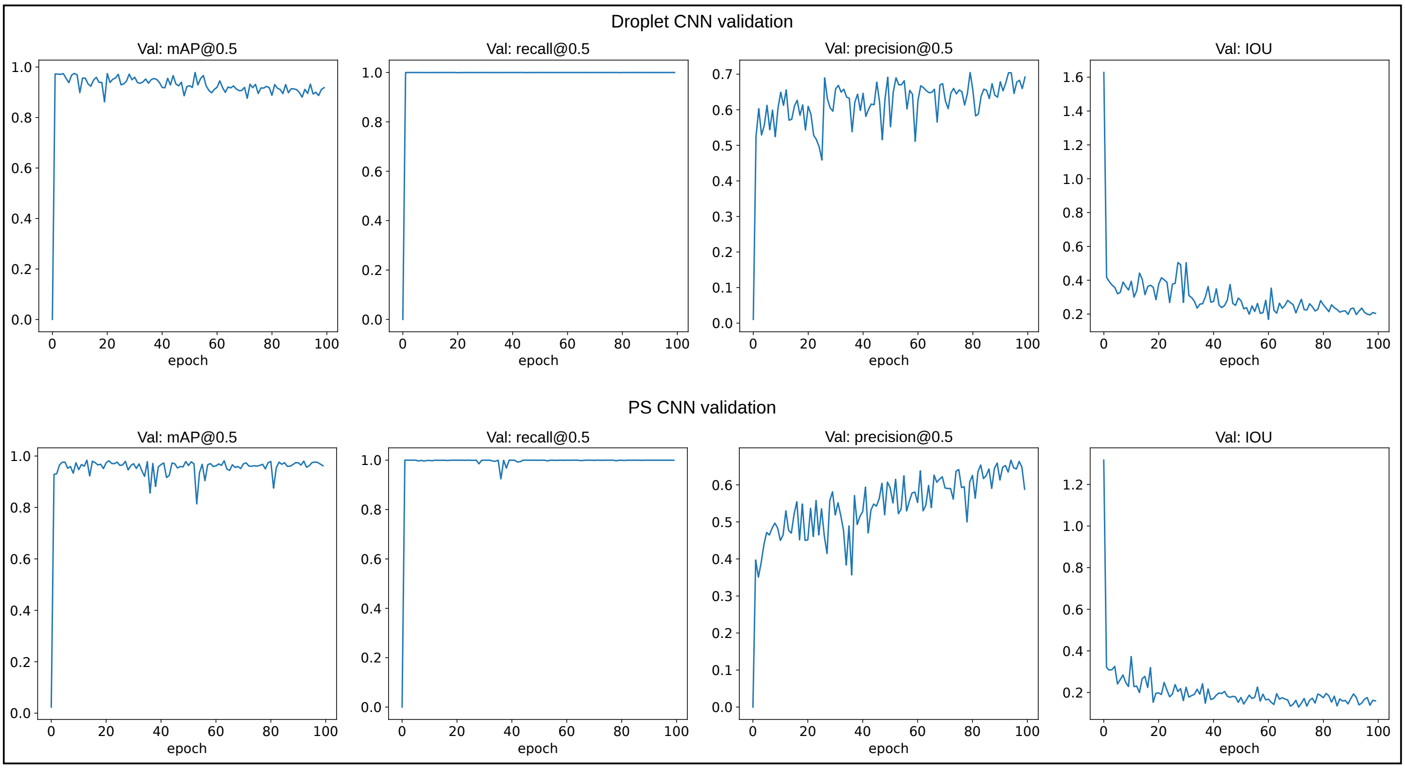


**Supplementary Figure 2. Validation parameters of the training of droplet detection CNN (top row) and PS detection CNN (bottom row).**

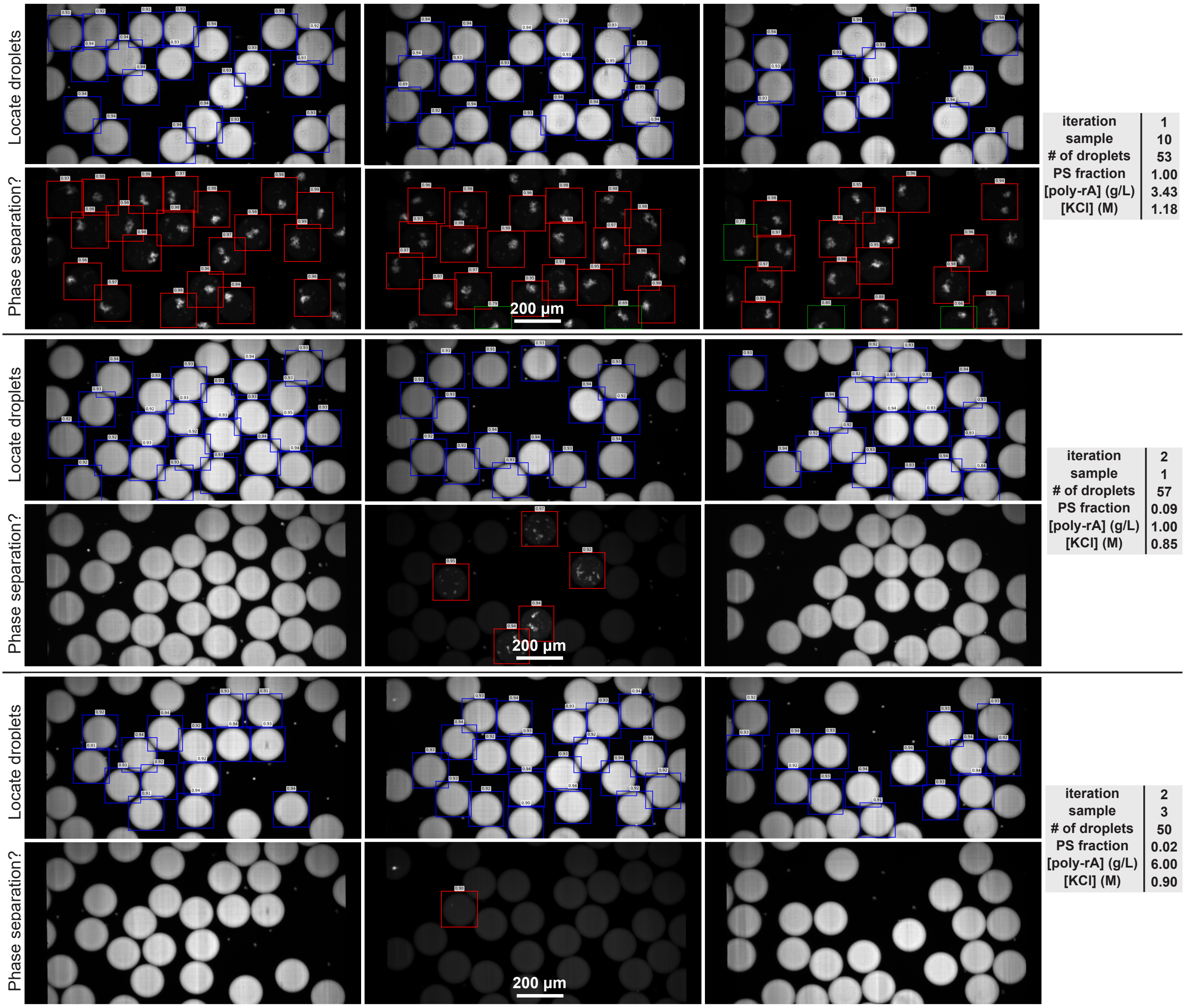


**Supplementary Figure 3. Images with analysis of the hybrid 3D experiment**. During the hybrid 3D experiment, droplets are imaged at at 647 ("Locate droplets") and 488 nm ("Phase separation?"). For 3 example samples, we show these images. Additionally, the neural network assignments (colored boxes and scores) are plotted on top. Droplets were searched for in the top images and are shown with blue boxes. Phase separated droplets were searched for in the bottom images and are indicated by red boxes. Green boxes indicate a (phase separated) droplet was found with lower accuracy (score). For each located droplet, the script checks if at the corresponding location in the 488 image a phase separated droplet was found. The scale bars apply to all images. Additional images with analysis are shown in supplementary figure 4.

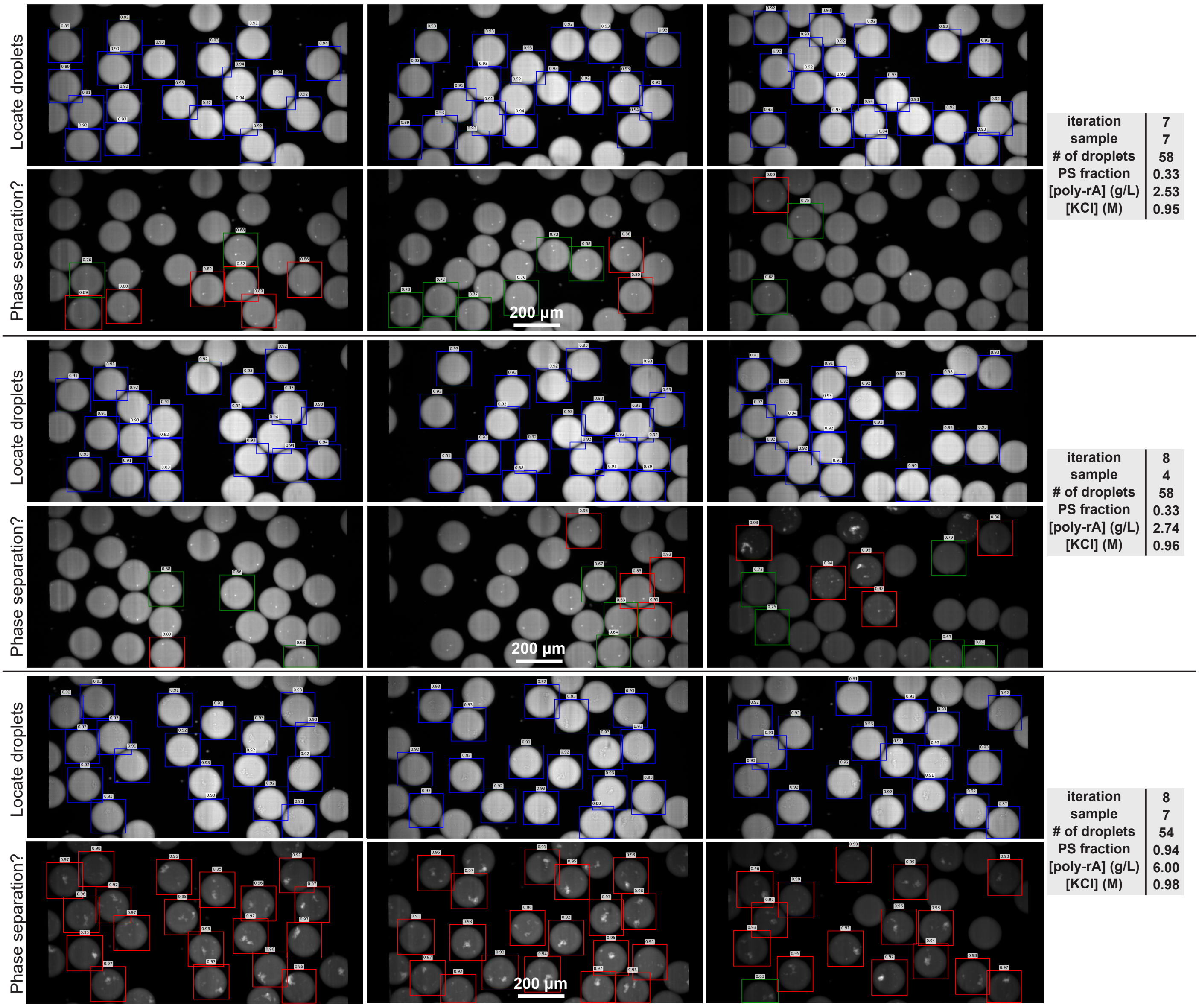


**Supplementary Figure 4. Additional images with analysis of the hybrid 3D experiment**. During the hybrid 3D experiment, droplets are imaged at at 647 ("Locate droplets") and 488 nm ("Phase separation?"). For 3 example samples, we show these images. Additionally, the neural network assignments (colored boxes and scores) are plotted on top. Droplets were searched for in the top images and are shown with blue boxes. Phase separated droplets were searched for in the bottom images and are indicated by red boxes. Green boxes indicate a (phase separated) droplet was found with lower accuracy (score). For each located droplet, the script checks if at the corresponding location in the 488 image a phase separated droplet was found. The scale bars apply to all images.

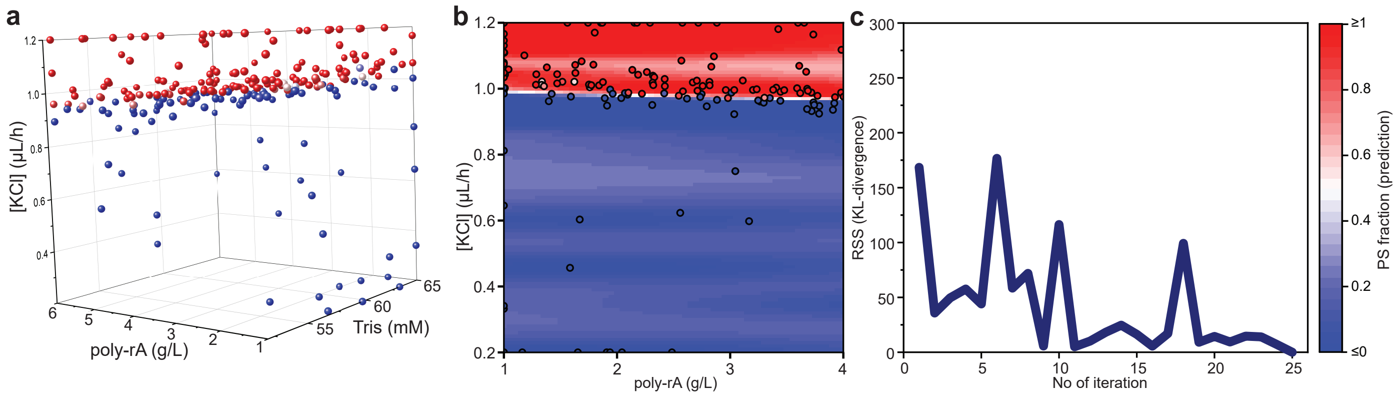


**Supplementary Figure 5. Reference diagram for the 3D experiment**. 250 datapoints were acquired in hybrid mode for the same poly-rA batch as Fig. 2.

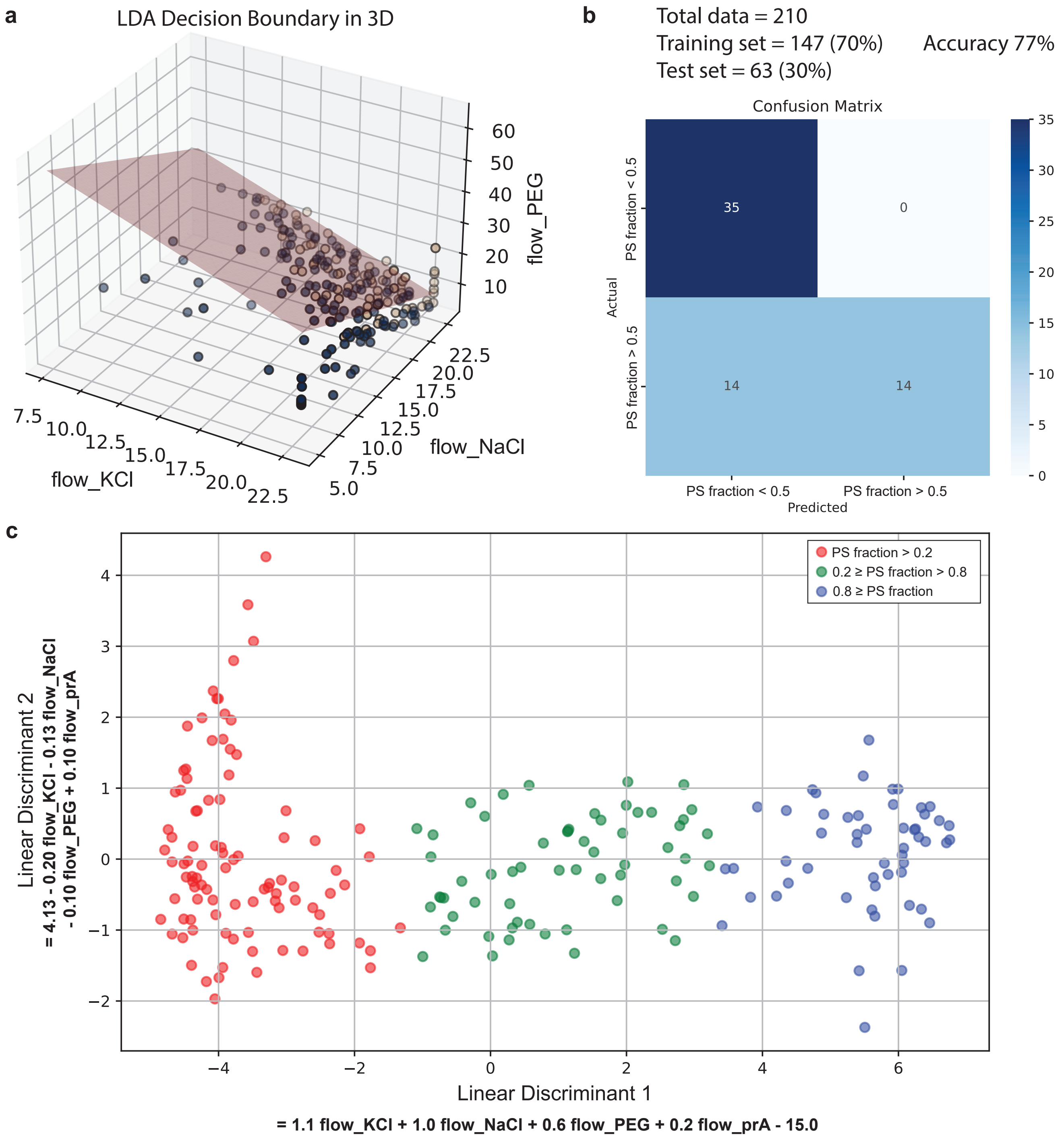


**Supplementary Figure 6. Further LDA analysis of the 4D experiment**. LDA analysis is performed (Figure 3b) to determine the order of most important variables [3]. **a** When plotting the data (beige is PS fraction $<0.5$, blue is is PS fraction $>0.5$) and the obtained boundary (red sheet) in 3D, we see that it is positioned approximately in the right location. Note that LDA is a tool for data interpretation, not a manner of fitting the phase boundary, which is often not a linear sheet, but a curved line/sheet/area. **b** Confusion matrix for the LDA decision boundary. PS fraction $< 0.5$ is correctly predicted using the boundary, but PS fraction $> 0.5$ have a 50 chance to be wrongly assigned. This is another indication that this method is unsuitable for locating the phase boundary. **c** LDA analysis can be performed on other groups than above or below 0.5 PS fraction. Here, 3 groups are separated based on wether the fraction is below 0.2 (red), between 0.2 and 0.8 (green) or above 0.8 (blue). Linear discriminant 1 and 2 both find KCl>NaCl>PEG>poly-rA as the order of importance for the separation. Note the additional use 2nd linear discriminant does not significantly improve the separation of the 3 datasets in comparison to use the use of the 1st linear discriminant. As such, the Bayes boundary as computed for the 1 linear discriminant is shown and used in figure 3b.

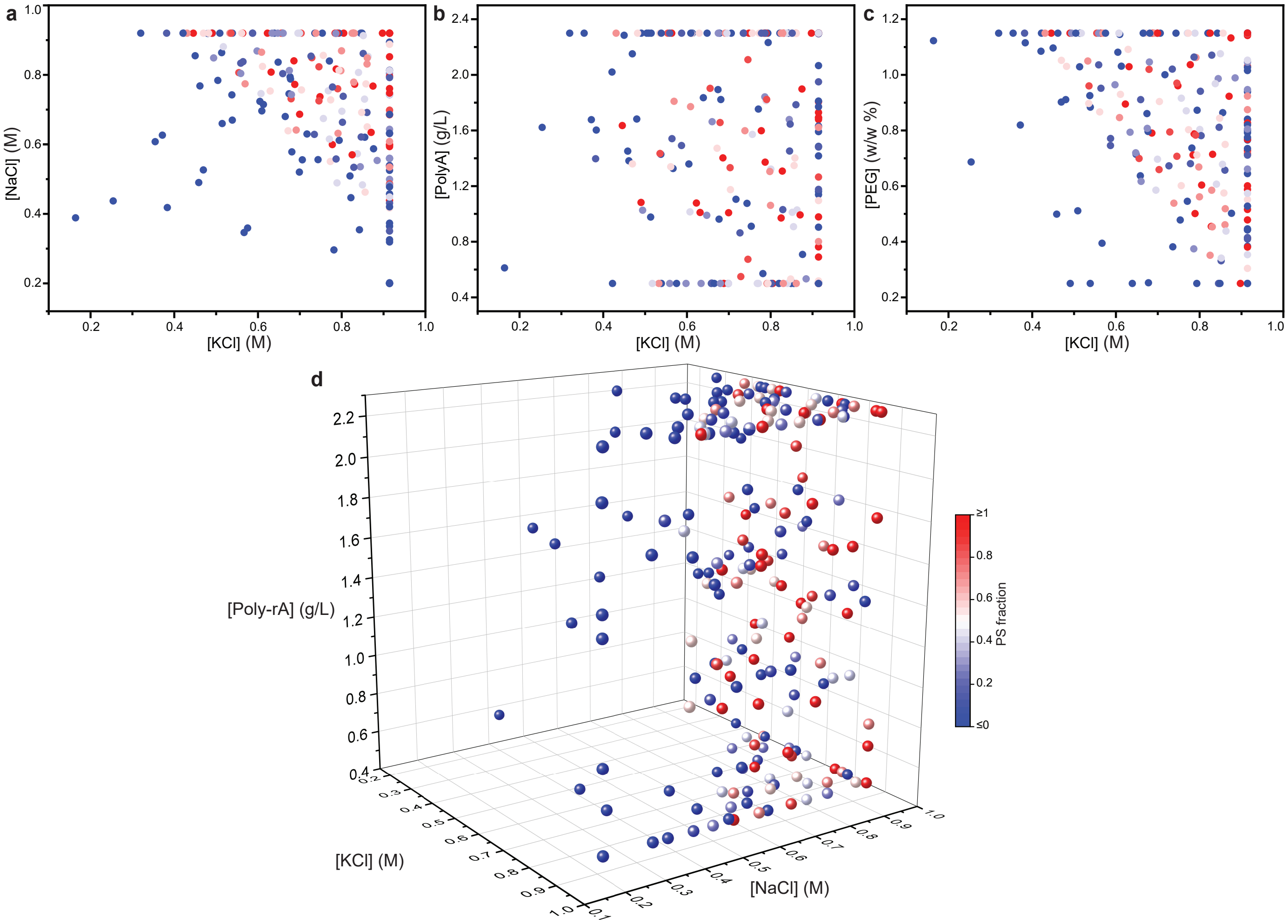


**Supplementary Figure 7. Projections of the 4D phase diagrams**. To plot the 4D experiment, we need to reduce the number of variables shown (create a projection). In figure 3d, this projection is chosen based on the LDA results. Here, projections of the 4D phase diagram are shown with on the axis **a** [NaCl] and [KCl], **b** [poly-rA] and [KCl], **c** [PEG] and [KCl], and **d** [poly-rA], [NaCl] and [KCl]. Note that in contrast to figure 3d, in these projections it is not possible or difficult to observe a phase boundary. As such, performing LDA is an important step when visualizing and interpreting the 4D data.

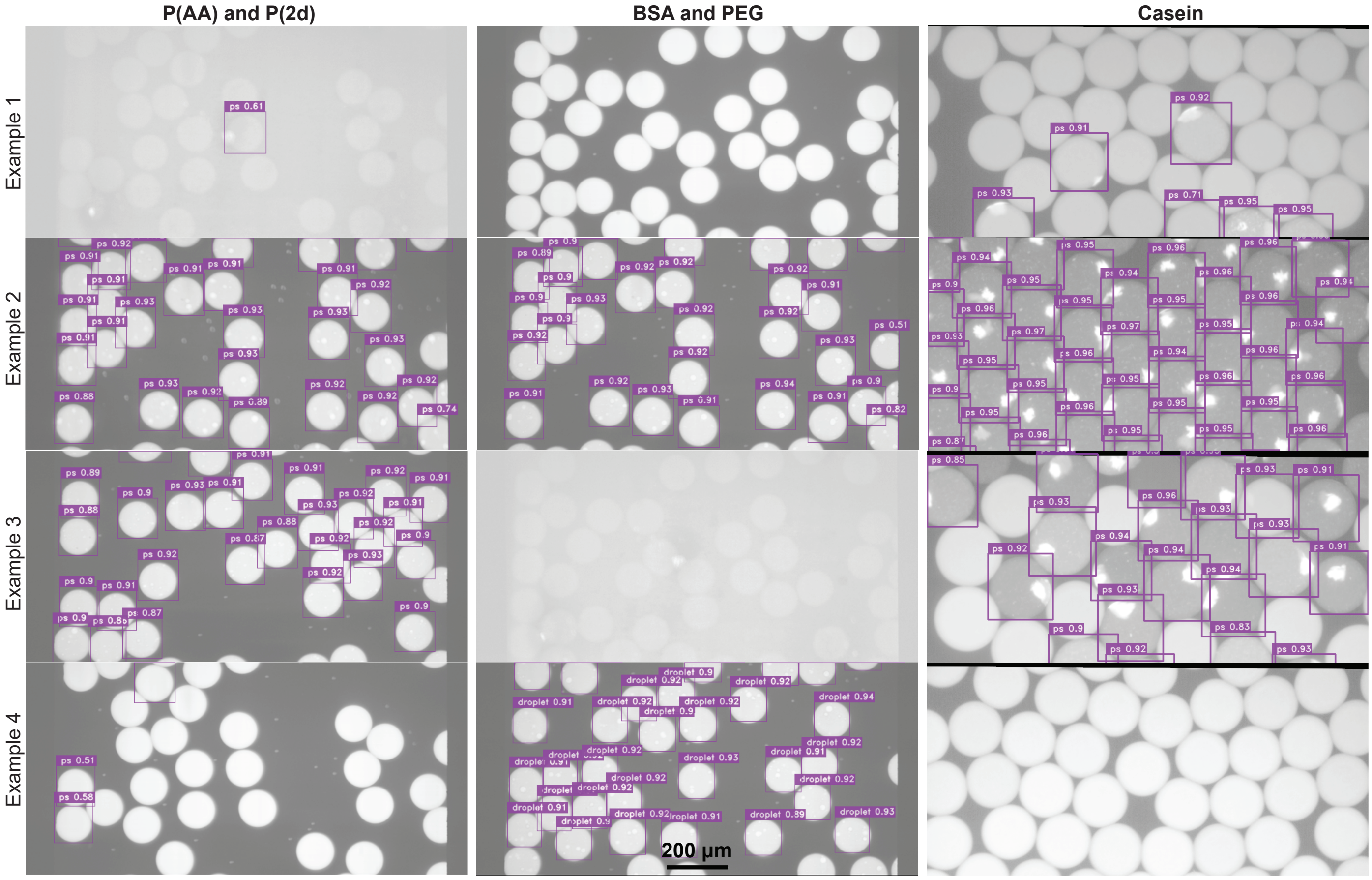


**Supplementary Figure 8. Phase separated droplet CNN analysis of samples containing various materials.**. The CNN which locates phase separated droplets should recognize droplets containing condensates and aggregates or various sizes and morphologies. Here, we confirm this is true by analyzing images with droplets containing various concentrations of poly(acrylic acid) , P(AA), and poly(2-[dimethylamino]ethyl methacrylate), P(2d); Bovine serum albumine, BSA, and Polyethylene glycol 8k, PEG; and casein protein and $Ca^{2+}$. Indeed, the CNN quite accurately identifies the phase separated droplets while not marking the homogeneous droplets. The CNN performs well for various droplet sizes. Please note that these images have been log-normalized and converted to 8-bit. The scale bar applies to all images.

# Supplementary references


1. Deci AI. *YOLONAS Documentation — SuperGradients* `https://github.com/Deci-AI/super-gradients/blob/master/YOLONAS.md`. Accessed: 2025-04-10. 2023.
2. Erkamp, N. A. *et al.* Multidimensional protein solubility optimization with an ultrahigh-throughput microfluidic platform. *Analytical Chemistry* **95,** 5362–5368 (2023).
3. Balakrishnama, S. & Ganapathiraju, A. Linear discriminant analysis-a brief tutorial. *Institute for Signal and information Processing* **18,** 1–8 (1998).
